# Supplementary material for: Endothelial Dysfunction Following Enhanced TMEM16A Activity in Human Pulmonary Arteries
Source: Cells. 2020 Aug 28;9(9):1984. doi: 10.3390/cells9091984 (PMC7563136; doi:10.3390/cells9091984)
Supplement: Supplementary file 1 [file cells-09-01984-s001.zip › Paper blueprint_supplement_revised.pdf]

Supplementary Figure 1

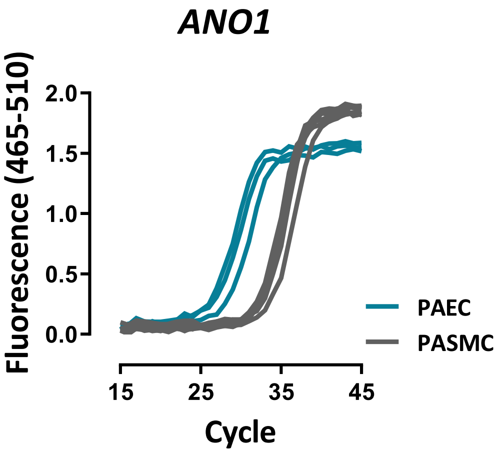

(a)

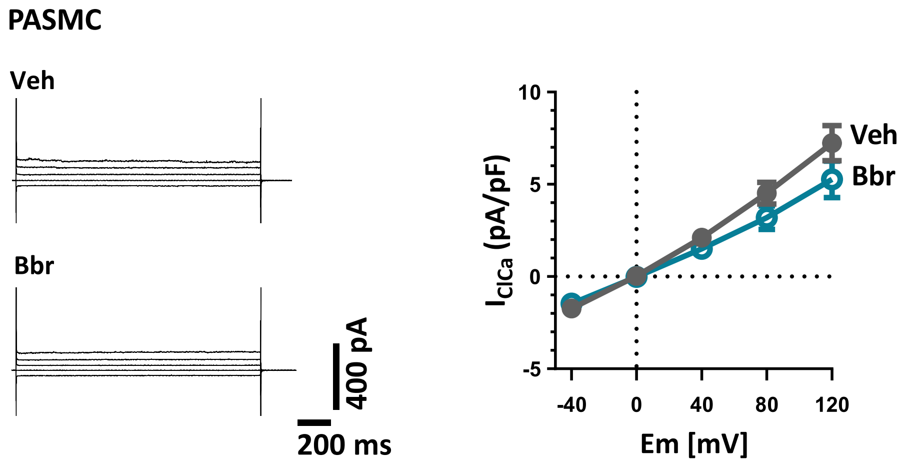

(b)

Figure S1. TMEM16A in pulmonary arterial endothelial and smooth muscle cells (PAECs and PASMCs respectively). (**a**) Gene expression of ANO1 in donor PAECs. (**b**) Representative whole-cell Ca^{2+}-activated Cl^- current (I_{ClCa}) traces (left) and normalized current-voltage (I-V) relationships (right) measured with voltage clamp in PASMCs of healthy donors showing the effect of benzbromarone (Bbr). Figures were generated with n = 5 cells from N = 2 healthy donors.

Supplementary Figure 2

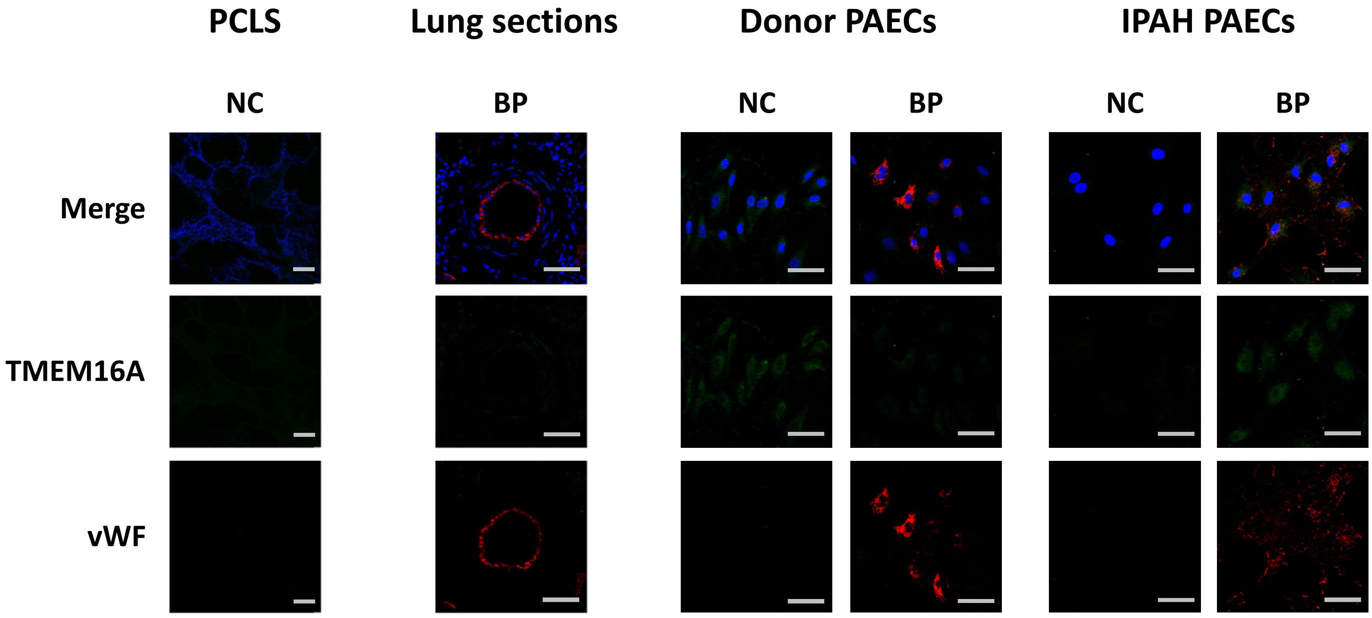

(a) (b) (c) (d)

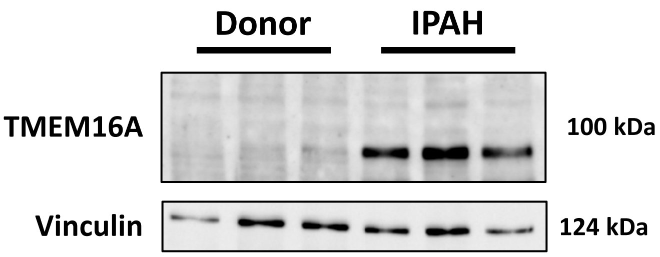

(e)

Figure S2. Control staining for detection of TMEM16A in PAECs. Immunofluorescence staining of 3D precision cut lung slices. (**a**) PCLS, (**b**) lung sections and (**c**) PAECs obtained from healthy donor lungs and (**d**) patients suffering from IPAH (NC=negative control lacking primary antibodies, BP = antibody blocking peptide, scale bar = 50 µm for PCLS, 50 µm for PAECs and 50 µm for lung sections). (**e**) Western blot comparing TMEM16A expression in donor and IPAH PAECs. Figures were generated with N = 3 samples for both groups.

Supplementary Figure 3

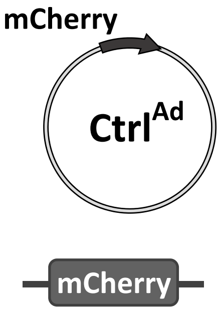

(**a**)

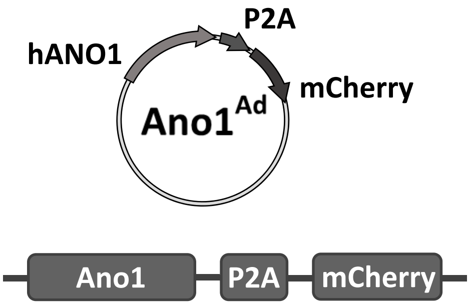

(**b**)

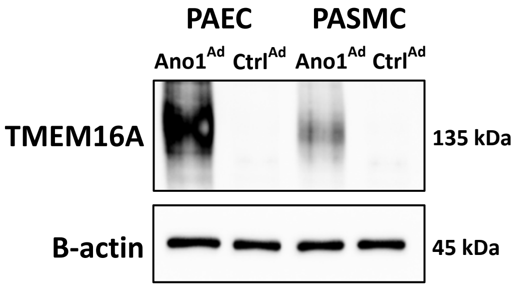

(**c**)

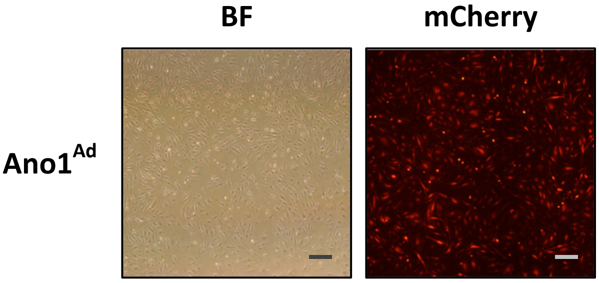

(**d**)

Figure S3. Adenovirus-induced expression of TMEM16A in human PAECs. Two adenoviruses were acquired from Vectorbuilder Inc. (**a-b**) Ctrl^{Ad} and Ano1^{Ad} expressing either mCherry (a) or mCherry connected to Ano1 via a self-cleaving peptide P2A respectively (b). (**c**) Western blot displaying TMEM16A expression in donor PAECs and PASMCs. (**d**) mCherry was chosen for the possibility of tracing the efficiency of the infection seen here with PAECs (scale bar = 200 μm).

Supplementary Figure 4

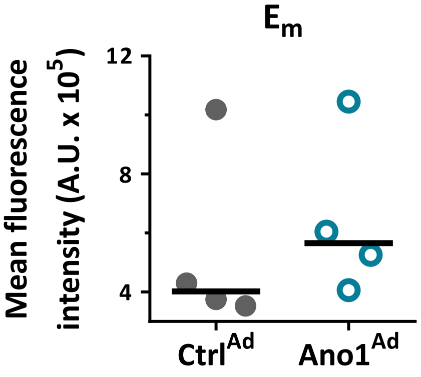

(a)

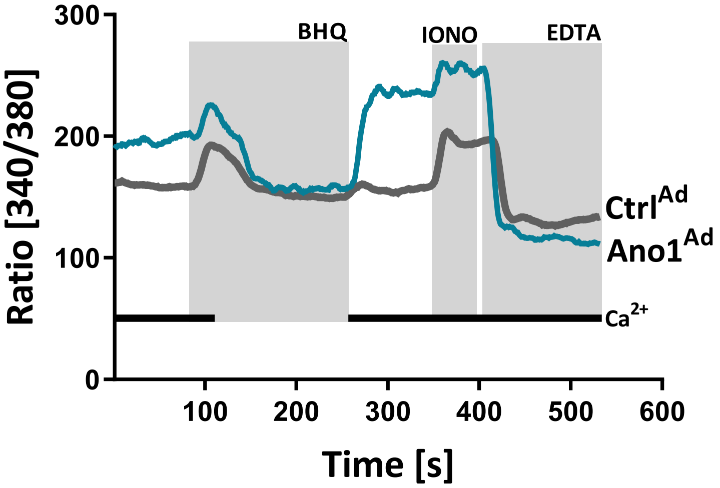

(b)

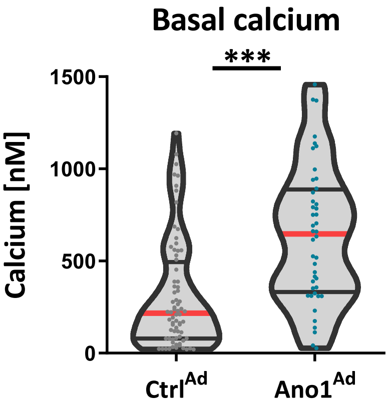

(c)

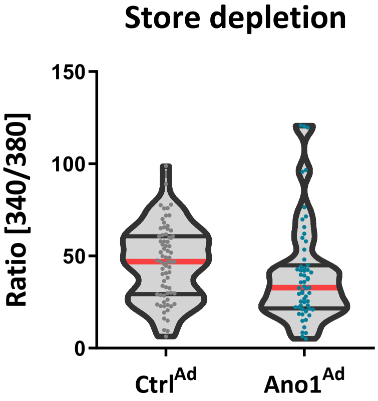

(d)

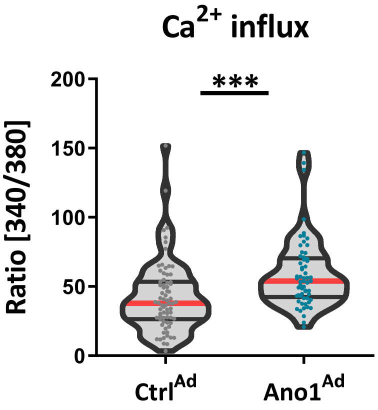

(e)

Figure S4. TMEM16A-mediated membrane depolarization disrupts Ca^{2+} dynamics of human PASMCs. (**a**) Fluorometric measurements indicating relative resting membrane potential (E_m) shift of donor PASMCs infected with $Ctrl^{Ad}$ or $Ano1^{Ad}$ using $DiBAC_4(3)$ dye. (**b**) Representative traces depict changes in intracellular Ca^{2+} measured in PASMCs transfected with $Ctrl^{Ad}$ or $Ano1^{Ad}$. (**c-e**) The effect of TMEM16A overexpression on cytosolic baseline Ca^{2+} concentration ($[Ca^{2+}]_i$), store depletion and Ca^{2+} influx using Fura-2 in donor PASMCs infected with $Ctrl^{Ad}$ or $Ano1^{Ad}$. (BHQ = butylhydroquinone). Figures were generated with 44-72 cells from N = 3 healthy donors. * $p < 0.05$, *** $p < 0.001$, unpaired t-test.

Supplementary Figure 5

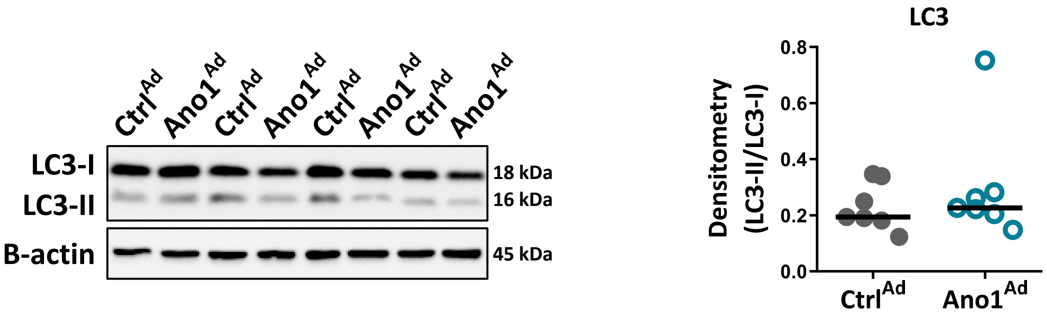

(a)

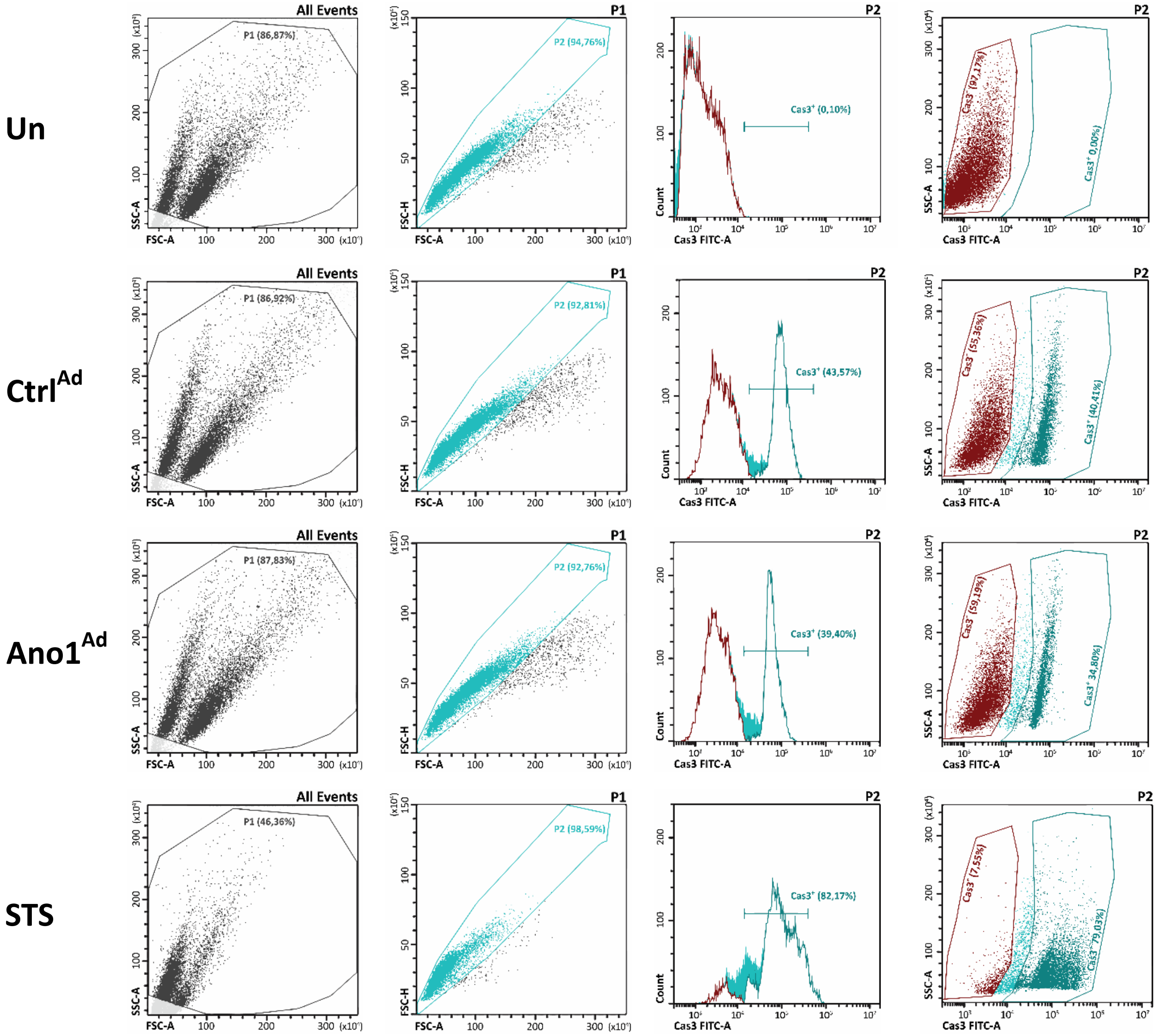

(b)

Figure S5. TMEM16A-overexpressing PAECs retain intact autophagy and apoptosis pathways. (**a**) Western blot of PAECs infected with TMEM16A-overexpressing $Ano1^{Ad}$ and control $Ctrl^{Ad}$ showing autophagy marker LC3. Figures were generated with n = 7 samples from 3 different donors. (**b**) Gating strategy for Cas3/Cas7 apoptosis assay including unstained control and positive control samples incubated with 10 µM staurosporin (STS) for 24 h.

Supplemental Figure 6

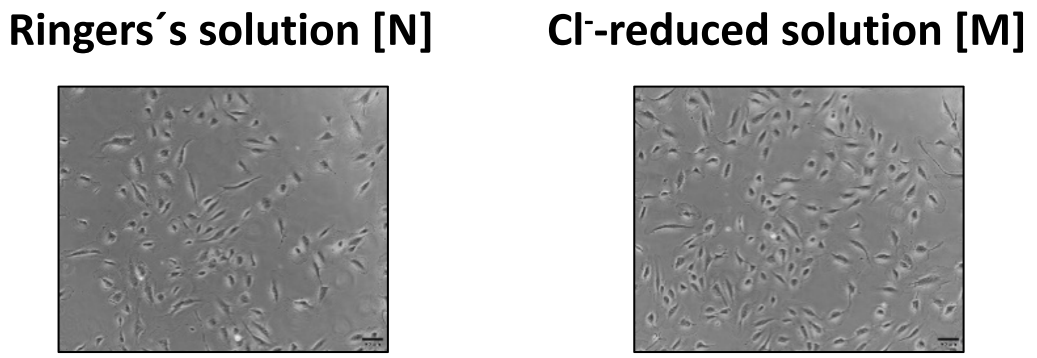

(a)

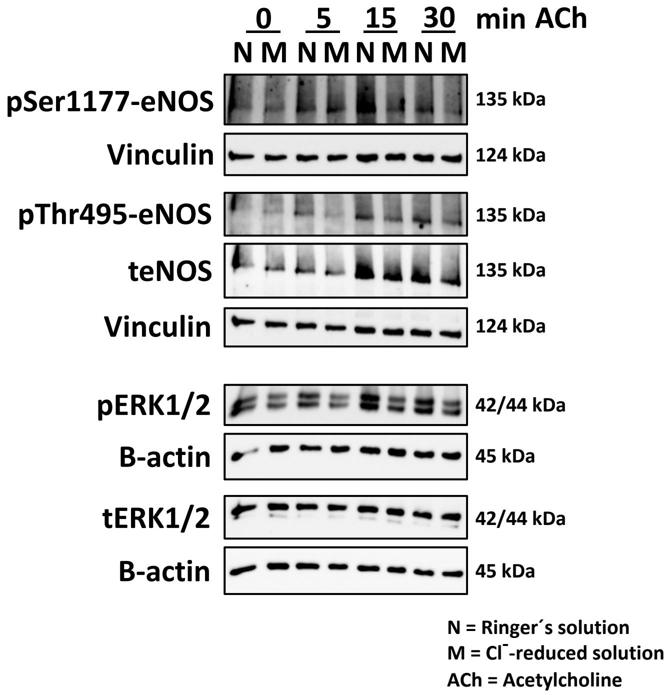

(b)

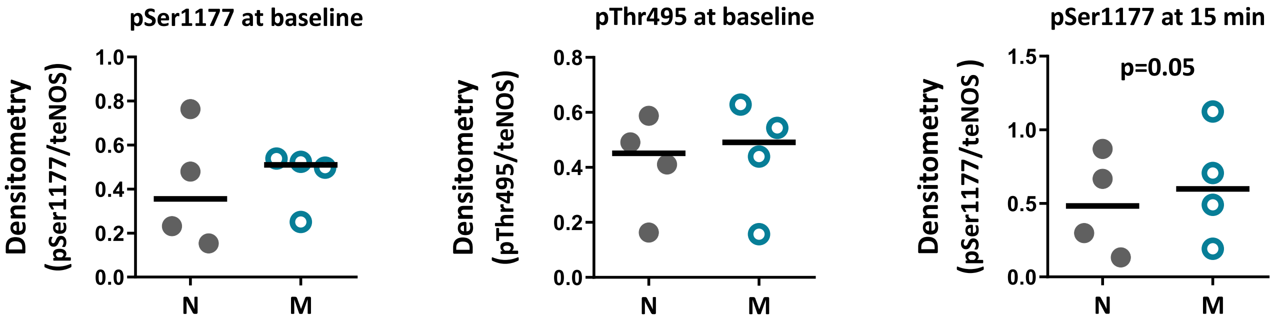

(c)

Figure S6. The effect of chronic intracellular Cl^- reduction on eNOS activation. (**a**) Bright-field pictures of donor PAECs incubated in either Ringer´s or Cl^--reduced solution for 24h before collecting the protein for further analysis (scale bar = 50µm). (**b**) Western blots showing Ach-induced changes in ERK1/2 and eNOS phosphorylation of $Ctrl^{Ad}$ and $Ano1^{Ad}$-infected donor PAECs. (**c**) Quantification of basal, non-induced level of eNOS phosphorylation at activatory Ser1177 sites as well as phosphorylation of Ser1177 15 minutes after ACh stimulation. Figures were generated with 4 samples. Ratio-paired t-test.

Supplementary Figure 7

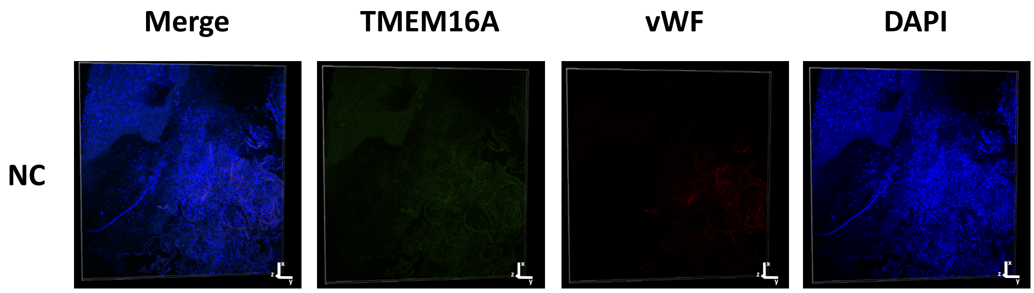

Figure S7. Immunofluorescence staining of 3D donor precision cut lung slices. Immunofluorescence staining of donor 3D precision cut lung slices (PCLS) (NC = negative control lacking primary antibodies; Width = 1257.93 µm, Height = 1257.93 µm,
