# Supplementary material for: Endothelial Dysfunction Following Enhanced TMEM16A Activity in Human Pulmonary Arteries
Source: Cells. 2020 Aug 28;9(9):1984. doi: 10.3390/cells9091984 (PMC7563136; doi:10.3390/cells9091984)
Supplement: Supplementary file 1 [file cells-09-01984-s001.zip › Skofic Maurer et al_Supplementary tables_revised.pdf]

Supplementary tables

Table S1. Patient characteristics *

Lung ID	Age (yr)	Sex (M/F)	mPAP (Hgmm)	Material used
Donor 1	16	M		PASMCs
Donor 2	47	M		PASMCs
Donor 3	50	F		PASMC
Donor 4	22	M		PASMCs
Donor 5	22	M		PASMCs
Donor 6	42	F		LT
Donor 7	58	M		LT
Donor 8	30	M		PASMCs
Donor 9	55	F		PASMCs
Donor 10	32	M		PASMCs
Donor 11	23	M		PASMCs
Donor 12	56	F		PASMCs
Donor 13	31	F		PASMCs
Donor 14	76	F		PASMCs
Donor 15	68	F		PASMCs
Donor 16	59	F		PAs, PAECs
Donor 17	48	F		PAs
Donor 18	45	M		PAs
Donor 19	54	M		LT
IPAH 1	25	F	69	LT
IPAH 2	13	M	56	PAECs
IPAH 3	41	F	71	PAECs
IPAH 4	21	M	90	PAECs
IPAH 5	38	F	39	PAECs

*Age, sex and mean pulmonary arterial pressure (mPAP) of healthy lung transplant donors and recipient IPAH patients. The materials used from each lung (PAECs: pulmonary arterial endothelial cells; PASMCs: pulmonary arterial smooth muscle cells, LT: lung tissue) are shown.

Table S2. Cells acquired from Lonza *

Cell ID	Age (yr)	Sex (M/F)
28032	51	M
28074	67	F
21292	21	M
21304	45	F
28343	52	F
28627	57	M
18664	63	M
15685	45	M
33756	34	F
27930	21	M
33941	48	M
35049	33	F

*Age and sex of donor PAECs acquired from Lonza.

Table S3. Primer sequences *

Gene	Acc. Number	Forward primer	Reverse primer	Product length (bp)
ANO1	XM_011545121.1	CACGATGAGGGTCAACGAGA	ATAAGGAGTTCAGCAGCGTG	128

*Exon-exon junction spanning primer sequences multiplying all splice variants of human TMEM16A.

Table S4. Antibodies used in western blot (WB) and immunofluorescence staining (IF) *

Antibody	Company	Catalogue number	Experiment	Dilution
Fibronectin	Abcam, UK	#ab23750	marker confirmation (isolated PAECs)	1:200
Smooth muscle myosin heavy chain		#ab53219	marker confirmation (isolated PAECs)	1:200
TMEM16A		#ab53212	WB (PAECs,, PASMCs, human lung homogenate, human pulmonary arteries)	1:1000
TMEM16A	Alomone Labs, Israel	#ACL-011	IF (PCLS, lung cuts, PAECs)	1:100
eNOS	BD Biosciences, USA	#610296	WB (PAECs)	1:1000
CD31		#550274	marker confirmation (isolated PAECs)	1:200
Cyclin D1	Cell Signaling, USA	#2978S	WB (PAECs)	1:1000
ERK1/2		#9102S	WB (PAECs)	1:1000
HRP-linked a-mouse		#7076S	WB	1:1000 – 1:5000
LC3B		#2775S	WB (PAECs)	1:1000
p38		#9212S	WB (PAECs)	1:1000
pAkt (S473)		#4058S	WB (PAECs)	1:1000
pAkt (T308)		#9275S	WB (PAECs)	1:1000
PARP		#9542S	WB (PAECs)	1:500
pERK1/2 (T202/Y204)		#9101S	WB (PAECs)	1:1000
pp38		#9211S	WB (PAECs)	1:1000
pSAPK/JNK (T183/Y185)		#9251L	WB (PAECs)	1:1000
pSer1177 eNOS		#9571S	WB (PAECs)	1:1000
pThr495 eNOS		#9574S	WB (PAECs)	1:1000
SAPK/JNK		#9252S	WB (PAECs)	1:1000
tAKT		#9272S	WB (PAECs)	1:1000
Vimentin		#3932	marker confirmation (isolated PAECs)	1:200
Von Willebrand factor	Dako, USA	M0616	IF (lung cuts, PAECs)	1:100

Von Willebrand factor		A0082	IF (PCLS);<br>marker confirmation (isolated PAECs)	1:100<br>1:500
Beta-Actin	Santa Cruz, USA	SC-47778	WB (PAECs, PASMCs)	1:2000
PCNA		SC-7907	WB (PAECs)	1:1000
Vinculin		SC-25336	WB (PAECs, PASMCs, human lung homogenate, human pulmonary arteries)	1:1000
Smooth muscle actin	Sigma-Aldrich, Germany	#A2547	marker confirmation (isolated PAECs)	1:300
Alexa Fluor 555 a-rabbit	ThermoFisher Scientific, USA	#A31572	IF (Lung sections, PAECs)	1:500
Alexa Fluor 647 a-mouse		#A31571	Lung sections, PAECs	1:500
AlexaFluor 488 a-rabbit		#A21206	IF (PCLS)	1:500
HRP-linked a-rabbit		#31460	WB	1:1000

*Antibodies used for western blot and immunofluorescence staining analysis with corresponding dilutions.

Table S5. Solutions *

Solution	Abbreviation	Ingredients (mM)		Used
Ringer´s solution	N	KCl (5.5), NaCl (119), $CaCl_2$ (1.5), $MgCl_2$ (1), Glucose (20), $NaHCO_3$ (26), HEPES (10), Na_2HPO_4 (0.5), KH_2PO_4 (0.5)	pH adjusted to 7.4; 0.2 µm-filtered; supplemented with 0,2 % penicillin/streptomycin and 0,5 or 2 % fetal bovine serum (Osmolality: ~307 mOsm/kg·H_2O)	Nitric oxide measurements, Western blot
Cl^--reduced Ringer´s solution	M	KCl (2.75), NaCl (59.5), potassium gluconate (2.75), sodium gluconate (59.5), $CaCl_2$ (1.5), $MgCl_2$ (1), Glucose (20), $NaHCO_3$ (26), HEPES (10), Na_2HPO_4 (0.5), KH_2PO_4 (0.5)	pH adjusted to 7.4 using NaOH; 0.2 µm-filtered; supplemented with 0,2 % penicillin/streptomycin and 0,5/2 % fetal bovine serum (Osmolality: ~302 mOsm/kg·H_2O)	Nitric oxide measurements, Western blot
Physiological salt solution	PSS	KCl (5.5), NaCl (140.5), $CaCl_2$ (1.5), $MgCl_2$ (1), Glucose (10), HEPES (10), Na_2HPO_4 (0.5), KH_2PO_4 (0.5)	pH adjusted to 7.4 using NaOH	Wire Myography, Ca^{2+} imaging
Physiological salt solution with isotonic replacement of NaCl by KCl	KPSS	KCl (120), NaCl (120), $CaCl_2$ (1.5), $MgCl_2$ (1), Glucose (10), HEPES (10), Na_2HPO_4 (0.5), KH_2PO_4 (0.5)	pH adjusted to 7.4 using NaOH	Wire Myography
PSS without Ca^{2+}		KCl (5.5), NaCl (140.5), $MgCl_2$ (1), Glucose (10), HEPES (10), Na_2HPO_4 (0.5), KH_2PO_4 (0.5), EGTA (1)	pH adjusted to 7.4 using NaOH	Ca^{2+} imaging
Lysis buffer	CHAPS[2]	NaCl (500), Tris-Hcl pH 7.5 (50), 5 % glycerol, 2 % CHAPS, 2 % sodium deoxycholate, 1 % sodium dodecyl sulfate	Supplemented with protease- and phosphatase inhibitors	Protein collection
TBS-T	TBS-T	Tris-HCl (5), NaCl (150), 0.4 % Tween 20	pH adjusted to 7.5	Western blot
Bath solution I		NaCl (150), $CaCl_2$ (1), $MgCl_2$ (1), glucose (10), HEPES (10)	pH adjusted to 7.4 using NaOH	Patch-Clamp
Bath solution II		NaCl (140), $CaCl_2$ (1), $MgCl_2$ (1), TEA-Cl (10),	pH adjusted to 7.4 using NaOH	Patch-Clamp

		glucose (10), HEPES (10)		
Pipette solution		CsCl (110), TEA-Cl (20), $CaCl_2$ (4.68), $MgCl_2$ (1), HEPES (10), EGTA (5), Na_2ATP (1)	pH adjusted to 7.2 using NaOH;<br>free Ca^{2+} concentration was 2 μM (MaxChelator software)	Patch-Clamp
Cutting solution		$CaCl_2$ (1.8), $MgSO_4$ (0.8), KCl (5.4), NaCl (116.4), NaH_2PO_4 (1.2), glucose (16.7), $NaHCO_3$ (26.1), HEPES (25.2)	pH adjusted to 7.2 using NaOH	PCLS
Incubation solution		$CaCl_2$ (1.8), $MgSO_4$ (0.8), KCl (5.4), NaCl (116.4), NaH_2PO_4 (1.2), glucose (16.7), $NaHCO_3$ (26.1), HEPES (25.2), sodium pyruvate (0.5), MEM-amino acid mixture (1:50), MEM-vitamins mixture (1:100), L-glutamine (1)	pH adjusted to 7.2 using NaOH;<br>penicillin (100 U/mL) / streptomycin (100 μg/mL)	PCLS

*Solutions developed in the course of this study.

Table S6. Materials *

Material	Company	Catalogue number
Seahorse XFp Cell Mito Stress Test Kit	Agilent technologies	103010-100
DMSO	AppliChem	A3672,0250
Low melting agarose	Bio-Rad, USA	1613111
[^{3}H]-thymidine	BIOTREND Chemikalien, Germany	ART-0178A-1/A306756
FCS	Biowest, France	S1810-500
384 Well PCR Platte	Biozym, Germany	711225X
Blue S'Green qPCR Kit		331416XL
CaCl2	Carl-Roth, Germany	5239.2
NaCl		3957.2
HEPES		HN78.2
Bis-Acrylamid, 30 %, Rotiphorese-Gel		3029.1

Dispase	Corning, USA	354235
Adenoviruses Ctrl^{Ad} and Ano1^{Ad}	Cyagen Biosciences, USA	hAno1 acc-number: NM_018043.5
Dako Target Retrieval Solution pH 9.0	Dako, Denmark	S236784
Dako Fluorescent mounting medium		S3023
Formaldehyde	Donauchem, Austria	A191483
U-44619	Enzo Life Sciences, USA	BML-PG023-0001
Na-dodecylsulfat (SDS) 10 %	Gatt-Koller, Germany	403030722
Sodium pyruvate	GE Healthcare, USA	S8636
ECL Start Western Blotting Detection Reagent		RPN3243
ECL Prime Western Blotting Detection Reagent		RPN2232
Filter Unit 0,2µm FP30/0		B285849
L-glutamine	Gibco, USA	8051103
MEM-amino acid mixture		11140035
MEM-vitamins mixture		11120037
2-well culture-inserts	Ibidi, Germany	80209
Fura-2AM	Invitrogen	F-1221
Ethanol 70 %	Lactan, German	T9132
CellEvent™ Caspase-3/7 Green Flow Cytometry Assay Kit	Life Technologies, USA	C10427
VascuLife Basal Medium	LifeLine Cell Technology, USA	LM-0002
VascuLife SMC Medium Complete Kit		LL-0014
Trypsin-EDTA	Lonza, Switzerland	CC-5012
TNS		CC-5002
EBM-2		LONCC-3156
EGM-2		LONCC-3162
In Vitro Angiogenesis Assay Kit	Merck, Germany	ECM625
Gelatine		9000-70-8
Novagen BCA Protein Assay Kit		A270811
Methanol		8395713
HBSS	PAA Laboratories, Austria	H15-009
DPBS	Pan Biotech, Germany	P04-36500
UniFilter-96 GF/C, White 96-well Barex Microplate with 1.2 µm poresize GF/C filter	Perkin Elmer, USA	6005174
TopSeal-A PLUS, Clear adhesive seal for microplates		6050185

Collagenase A	Roche Applied Science, Germany	10103586
CsCl	Reanal Laborvegyszer, Hungary	07020-0-01-25
T25 flasks	Sarstedt, Germany	83.3910.302
DNAse	Serva, Germany	18535.01
benzbromarone	Sigma-Aldrich, Germany	B5774-1G
KCl		P9333
NaH_2PO_4		S3139-250G
D-(+)-glucose		G8270-1KG
$NaHCO_3$		S5761-1KG
Acetylcholine		A6625.25G
Bovine Serum Albumin		A6003-10G
Triton X-100		T8787-100ML
Tris-HCl		C4706-2G
Glycine		G7126-5KG
Glycerol		G5516-100ML
Sodium deoxycholate		D6750-25G
Sodium bicarbonate		S5761
Sodiumphosphate monobasic monohydrate		71507
Potassium D-gluconate		G4500-1KG
TEA-Cl		T2265
Na_2ATP		A7699
Magnesium Sulfate Heptahydrate		M5921
D-Gluconic acid sodium salt		G9005-1KG
Western blotting membranes, nitrocellulose, pore size 0.45 µm		GE10600008
TEMED		T9281
Ammonium persulfate (APS)		A3678-25G
Tween 20		P7949
EGTA		E4378-100G
Ionomycin calcium salt		I3909
L-NAME		N5751-10G
Nunc-Immuno™ MicroWell™ 96 well polystyrene plates BLACK		137101
$DiBAC_4(3)$		D8189-25MG
Cell scrapers		8536585
Mix-n-Stain™ CF™ 633 Antibody Labeling Kit		MX633S50-1KT
T75 flasks		CC7682-4875

Dynabeads® CD31 Endothelial Cell	ThermoFisher Scientific, USA	11155D
Penicillin/Streptomycin (P/S)		15140-122
Pierce protease inhibitor tablets		A32953
Pierce phosphatase inhibitor tablets		A32957
SuperSignal West Femto Chemiluminescent Substrate		34095
Restore Plus Western Blot Stripping Buffer		A323654
StemPro Accutase Cell Dissociation Reagent		11599686
DAF-DM		D23842
8-well chambered cell culture slides		10162861
CHAPS	Tocris Bioscience, UK	3172
Vectashield mounting medium with DAPI	Vector Laboratories, UK	H-1200
Thick-walled borosilicate glass (Standard Glass Capillaries, 4 in., 1.2 / 0.68 OD/ID, Filament/Fire Polished)	World Precision Instruments, USA	1B120F-4

*Information regarding all the materials used during the course of this study.
